# P-2091. Potential Impact of Targeted Sequencing on Central Line-associated Bloodstream Infection (CLABSI) at Stanford Health Care, 2015–2024

**DOI:** 10.1093/ofid/ofae631.2247

**Published:** 2025-01-29

**Authors:** Guillermo Rodriguez-Nava, Goar Egoryan, Kyle Fu, John Shepard, Karen McIntyre, Mindy M Sampson, Jorge Salinas

**Affiliations:** Stanford University School of Medicine, Stanford, CA; Stanford University, Menlo Park, California; Stanford Health Care, Stanford, California; Stanford University, Menlo Park, California; Stanford Healthcare, Stanford, California; Stanford University, Menlo Park, California; Stanford University, Menlo Park, California

## Abstract

**Background:**

Central line-associated bloodstream infection (CLABSI) remains a key quality measure. However, the CLABSI surveillance definition attributes bloodstream infections in patients with central venous access devices to the central line if no other sources are found, potentially overestimating true line-related cases. Incorporating non-culture based microbiologic testing, such as next-generation sequencing, into the CLABSI surveillance definition may further skew these estimates. We aimed to review the organisms identified in 16S rRNA sequencing tests from blood samples to determine if they were typically associated with CLABSI.

**Methods:**

We retrieved all 16S rRNA sequencing orders from Stanford Hospitals & Clinics from May 2015– May 2024. We identified orders obtained from blood specimens and categorized those patients with and without central venous access devices. We compared the identified organisms with those included in the NSHN CLABSI definition (https://www.cdc.gov/nhsn/pdfs/pscmanual/17pscnosinfdef_current.pdf).

**Results:**

Of the 3,830 16S rRNA tests ordered, 225 were from blood specimens. Among these, 40% (65/225) had a central venous access device at some point during admission. Eight tests were positive: two from patients with a device during collection, identifying *Bartonella quintana* and *Ureaplasma urealyticum*—both not excluded from the NHSN definition. Other positives included two cases of *Borrelia hermsii*, *Paracoccus* spp., *Leptospira* spp., *Legionella* spp., and coagulase-negative *Staphylococcus*, with only the latter typically associated with central venous access device infection. Only organisms from the genera *Blastomyces*, *Histoplasma*, *Coccidioides*, *Paracoccidioides*, *Cryptococcus*, and *Pneumocystis* are excluded from the NHSN CLABSI definition.

**Conclusion:**

The increasing use of next-generation sequencing tests, such as 16S rRNA targeted sequencing, introduces unique scenarios where non-culturable organisms can be detected in blood specimens. There may be some risk of unfairly penalizing institutions for reporting organisms not associated with CLABSI. The list of organisms included in the NHSN CLABSI surveillance definition should be continuously updated to reflect the adoption of new non-culture-based testing methods.

**Disclosures:**

All Authors: No reported disclosures

